# PA32540 for the secondary prevention of cardiovascular disease in patients at risk for aspirin-associated gastric ulcers

**DOI:** 10.1586/14779072.2014.967214

**Published:** 2014-10-10

**Authors:** Danielle Duffy, Bridget Rooney, Suzanne Adams, David J Whellan

**Affiliations:** ^a^ Thomas Jefferson University, Philadelphia, PA 19107, USA

**Keywords:** aspirin, cardiovascular disease, omeprazole, polypill, secondary prevention

## Abstract

Prescribed in patients with a history of myocardial infarction, stroke, transient ischemic attack, coronary intervention or bypass surgery, aspirin is one of the medications most commonly used in the secondary prevention of cardiovascular diseases. It has become a mainstay of therapy after years of solid evidence supporting its efficacy in clinical trials. However, a number of risks and side effects accompany its benefits, including the notable risk of bleeding and gastrointestinal side effects. Numerous mechanisms have been proposed to attenuate these effects to promote adherence and to expand the population for which aspirin is a reasonable treatment option. A polypill or combination formulation that includes a proton pump inhibitor, a drug commonly prescribed alongside aspirin, is one potential avenue of therapy. One such combination pill, PA32540, has undergone Phase I and Phase III trials and shows promising safety and efficacy results in these preliminary trials.

## Overview & unmet needs

According to the American Heart Association, cardiovascular disease was listed as the underlying cause of death for 787,650 deaths in the USA in 2010, or one of every three deaths [1]. Aspirin, or acetylsalicylic acid (ASA), has been used in the secondary prevention of cardiovascular disease since the 1970s [2] and today is recognized as an integral treatment component in the secondary prevention of myocardial infarction (MI), stroke and transient ischemic attack (TIA). Aspirin is also recommended as a preventive therapy following coronary intervention or bypass surgery [3].

### Secondary prevention of cardiovascular disease

In the 1980s, the US FDA approved professional labeling indications for ASA in patients with prior MI and unstable angina. In January 1997, the FDA Nonprescription Drugs and Cardiovascular and Renal Drugs Advisory Committees recommended expansion of the labeling indication to include women as well as men with prior TIAs and patients with prior occlusive stroke or chronic stable angina.

More recently, The Antiplatelet Trialists’ Collaboration overview analyzed results of randomized trials of antiplatelet therapy among 287 studies including 212,000 patients. This group concluded that ASA is protective in most types of patients at increased risk of occlusive vascular events, including those with an acute MI or ischemic stroke, unstable or stable angina, previous MI, stroke or cerebral ischemia, peripheral arterial disease or atrial fibrillation. Low-dose ASA (75–150 mg daily) is an effective antiplatelet regimen for long-term use, while an initial dose of 150 mg is more appropriate in acute settings [4]. After percutaneous coronary intervention (PCI), ASA is recommended with P2Y12 inhibitors to reduce the risk of stent thrombosis [3]. Long-term ASA use (defined as ≥2 years) has been shown to reduce the risk of vascular events by 12% compared with patients administered placebo [5]. A recent meta-analysis of randomized trials estimated that the number needed to treat with ASA to prevent one death from any cause was 67 [6].

Despite these and numerous other studies showing the clinical efficacy of ASA use, clinical effectiveness is hindered substantially by underuse of these medications. Past studies have estimated ASA compliance to range from 72 to 92% [7]. Among patients who were prescribed ASA following hospitalization for an acute MI, patient adherence to the regimen was only 10% after 5 years, compared with 17% for statins, 31% for ACE inhibitors and 36% for β-blockers [8]. Patients who discontinue their ASA use are at greater risk of another cardiac event and have an increased risk of coronary heart disease-related death compared with those that continue ASA [7].

A number of concerns may contribute to poor compliance with ASA therapy, including difficulties with polypharmacy, lack of understanding of the benefits of the therapy and unfavorable side effects. In addition, in contrast to statin and β-blocker therapy, which respectively show concrete health improvements through lowered lipid levels and blood pressure readings, patients do not receive any real-time feedback on the effectiveness of an ASA regimen and often experience no symptoms from ASA non-compliance unless they experience another cardiovascular event. The most common side effects reported among ASA users include gastrointestinal effects, which may range from mild gastrointestinal upset to severe gastric bleeding. Patient characteristics that increase the risk of major gastrointestinal effects include age >55 years, history of gastric ulcers or upper gastrointestinal bleeding, non-steroidal anti-inflammatory drug (NSAID) use and *Helicobacter pylori* infection [9]. In one nationwide French survey, 15% of patients taking low-dose ASA therapy experienced gastrointestinal side effects, and 12% of this subset indicated that these side effects impacted their compliance to ASA therapy [10].

Evidence suggests that low-dose ASA may be just as effective as high-dose ASA in reducing cardiovascular risk. The Percutaneous Coronary Intervention–Clopidogrel in Unstable Angina to Prevent Recurrent Events compared low (≤100 mg), moderate (101–199 mg) and high (≥200 mg) doses of ASA and found that low doses were as effective as moderate and high doses in preventing cardiovascular death, MI and stroke. The occurrence of bleeding was more frequent in the high-dose group compared with the low-dose group (3.9 vs 1.9%; hazard ratio: 2.05; 95% CI: 1.20–3.50; p = 0.009) [11]. Similarly, the Clopidogrel and Aspirin Optimal Dose Usage to Reduce Recurrent Events-Seventh Organization to Assess Strategies in Ischemic Syndromes trial assessed clopidogrel and ASA dosing. Patients were randomized to receive either high-dose (300–325 mg) or low-dose (75–100 mg) ASA in combination with clopidogrel. After 1 month of treatment, the difference in the rates of cardiovascular death and MI between the high- and low-dose group were not significant (4.2 vs 4.4%; p = 0.61), but the high-dose ASA group showed a small increase in gastrointestinal bleeding (0.4 vs 0.2%; p = 0.04) [12].

Current guideline recommendations advise ASA doses between 81 and 325 mg [3]. Reviews of actual physician prescribing practices estimate that a significant proportion of physicians prescribe doses at the higher end of this range, which may be associated with greater gastrointestinal toxicity. A study of the National Cardiovascular Registry’s Acute Coronary Treatment and Intervention Outcomes Network Registry-Get with the Guidelines reviewed the ASA prescribing practices from 221,119 patients with an MI across 525 US hospitals between January 2007 and March 2011. High-dose ASA (defined as 325 mg) was prescribed for 60.9% of patients at discharge and was prescribed for 73.0% of patients treated with PCI and 44.6% of patients managed medically. High-dose ASA was chosen even for those with a major in-hospital bleeding event (56.7%) and among those discharged on a combination of ASA, warfarin and thienopyridines (44.0%) [13]. Overall, an estimated 35% of patients taking ASA in the USA take a dose of 325 mg or greater [14].

Enteric-coated ASA (EC-ASA) has been suggested as one method to combat unfavorable gastrointestinal side effects by preventing ASA dissolution in the stomach and delaying release until it reaches the small intestine. A significant proportion of patients, however, continue to experience gastrointestinal bleeding while using EC-ASA [15]. Proton pump inhibitor (PPIs), on the other hand, have shown greater promise than EC-ASA in minimizing gastrointestinal side effects in several randomized trials [16]. Continuous PPI use has been associated with a lower risk of gastrointestinal ulcers or bleeding compared with intermittent or no PPI use [17]. However, some evidence suggests that physicians do not frequently prescribe PPI therapy to higher risk patients [18] and that patients struggle to adhere to this medication regimen when it is prescribed [19].

As polypharmacy has been one suspected cause of non-compliance, pills that combine ASA and a PPI may help patients to better adhere to their medication regimens [20], reduce patient frustration with polypharmacy and make ASA therapy safer for patients at high risk for gastrointestinal bleeding. Patients have indicated that they have a favorable impression and willingness to try combination pills [21], and combination pills may reduce costs.

**Figure 1.**
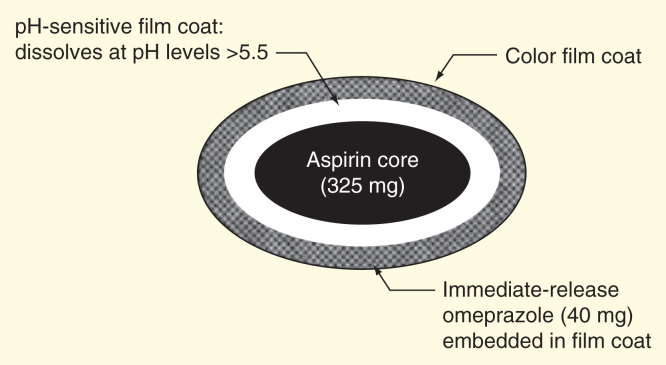
**PA32540 coordinated-delivery tablet [28].**

PA32540 is a novel combination pill of 325 mg of ASA surrounded by 40 mg of immediate-release omeprazole (Figure 1). PA32540, along with combination ASA-omeprazole pill PA8140 (ASA of 81 mg + 40 mg of omeprazole), is under development to combine ASA + PPI in a single tablet. PA32540 is reviewed in detail here (see Supplementary Appendix 1 [Supplementary material can be found online at www.informahealthcare.com/suppl/10.1586/14779072.2014.967214]) due to the greater potential for gastrointestinal and bleeding side effects with a higher ASA dose.

**Figure 2.**
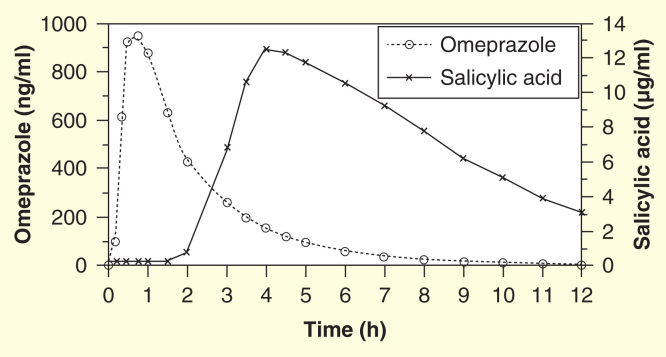
**The pharmacokinetic release profile of omeprazole and salicylic acid from PA32540 on day 7 [28].**

The tablet is a coordinated release tablet. Omeprazole is immediately released into the stomach upon ingestion and ASA is released when the pH >5.5. Figure 2 shows the release profile of the drug. A similar combination pill including ASA and pantoprazole is under development; however, the pill has undergone only Phase I testing and its results have not yet been made public [22].

## Introduction to the drug

### Aspirin: introduction to the drug

**Figure 3.**
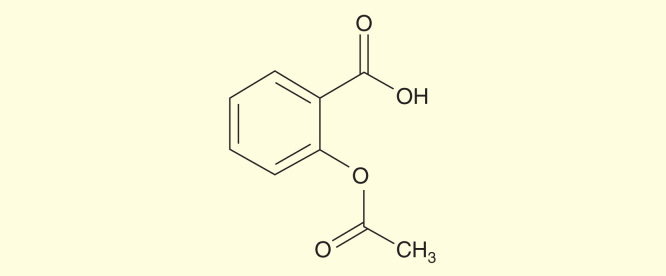
**The molecular structure of aspirin.**

The chemical name for ASA is acetylsalicylic acid. The molecular formula for acetylsalicylic acid is C_9_H_8_O_4_ (Figure 3). The molecular weight is 180.15742. When exposed to moisture, ASA hydrolyzes into salicylic and acetic acids, and gives off a vinegary-odor. It is highly lipid soluble and slightly soluble in water.

#### Pharmacodynamics

ASA is a weak organic acid. It is absorbed mainly in the stomach and upper small intestine. The main metabolite is 2-hydroxybenzoic acid (salicylic acid).

ASA has four separate pharmacodynamics effects on the body. These include:

- Anti-inflammatory effect which reduces stiffness and swelling;
- Analgesic effect which attenuates or alleviates pain;
- Antipyretic effect which lowers body temperature during fever;
- Antiplatelet effect which reduces platelet activation and thrombus formation.

ASA affects platelet aggregation by irreversibly inhibiting prostaglandin cyclo-oxygenase. This effect lasts for the life of the platelet and prevents the formation of the platelet aggregating factor thromboxane A2. ASA is a more potent inhibitor of both prostaglandin synthesis and platelet aggregation than other salicylic acid derivatives. The differences in activity between ASA and salicylic acid are thought to be due to the acetyl group on the ASA molecule. This acetyl group is responsible for the inactivation of cyclo-oxygenases via acetylation. Non-acetylated salicylates do not inhibit this enzyme and have no effect on platelet aggregation.

At somewhat higher doses, ASA reversibly inhibits the formation of prostaglandin I_2_ (prostacyclin), which is an arterial vasodilator and inhibits platelet aggregation. At higher doses, ASA is an effective anti-inflammatory agent, partially due to inhibition of inflammatory mediators via cyclo-oxygenase inhibition in peripheral tissues. *In vitro* studies suggest that other mediators of inflammation may also be suppressed by ASA administration, although the precise mechanism of action has not been elucidated. It is this non-specific suppression of cyclo-oxygenase activity in peripheral tissues following large doses that leads to its primary side effect of gastric irritation.

#### Pharmacokinetics

In general, immediate-release ASA is well and completely absorbed from the GI tract. Following absorption, ASA is hydrolyzed to salicylic acid with peak plasma levels of salicylic acid occurring within 1–2 h of dosing. The rate of absorption from the GI tract is dependent upon the dosage form, the presence or absence of food, gastric pH (the presence or absence of gastrointestinal antacids or buffering agents) and other physiologic factors.

Salicylic acid is widely distributed to all tissues and fluids in the body including the CNS, breast milk and fetal tissues. The highest concentrations are found in the plasma, liver, renal cortex, heart and lungs. The protein binding of salicylate is concentration-dependent, that is, non-linear. At low concentrations (<100 µg/ml), approximately 90% of plasma salicylate is bound to albumin, while at higher concentrations (>400 µg/ml), only about 75% is bound.

**Figure 4.**
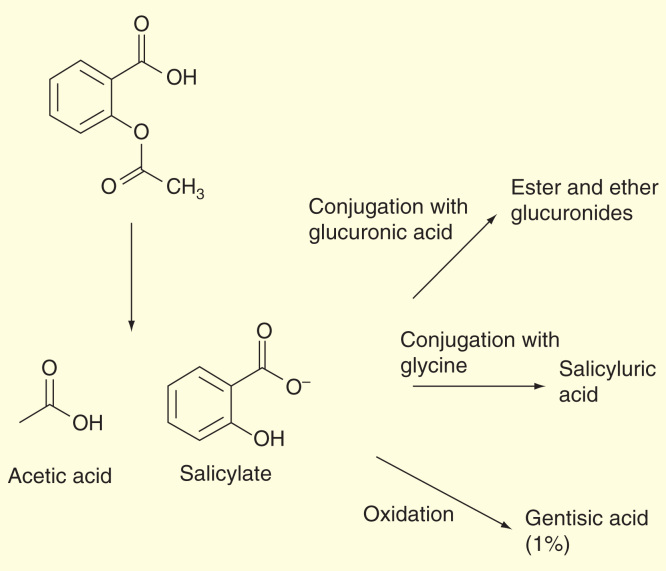
**The pharmacokinetics of aspirin.**

ASA is rapidly hydrolyzed in the plasma to salicylic acid such that plasma levels of ASA are essentially undetectable 1–2 h after dosing. Salicylic acid is primarily conjugated in the liver to form salicyluric acid, a phenolic glucuronide, an acyl glucuronide and a number of minor metabolites (Figure 4). Salicylic acid has a plasma half-life of approximately 6 h. Salicylate metabolism is saturable and total body clearance decreases at higher serum concentrations due to the limited ability of the liver to form both salicyluric acid and phenolic glucuronide. The elimination of salicylic acid follows zero order pharmacokinetics; (i.e., the rate of drug elimination is constant in relation to plasma concentration). Renal excretion of unchanged drug depends upon urine pH. As urinary pH rises above 6.5, the renal clearance of free salicylate increases from <5 to >80%. Following therapeutic doses, approximately 10% is found excreted in the urine as salicylic acid, 75% as salicyluric acid and 10% phenolic and 5% acyl glucuronides of salicylic acid.

### Omeprazole: introduction to the drug

**Figure 5.**
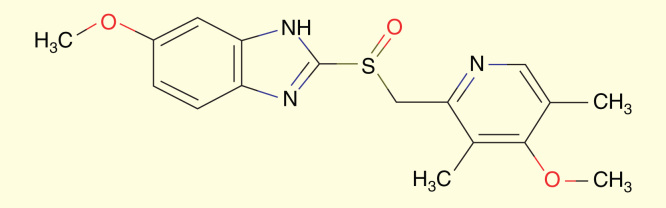
**The molecular structure of omeprazole.**

Omeprazole is a highly effective inhibitor of gastric acid secretion used in the therapy of stomach ulcers. Omeprazole belongs to a class of anti-secretory compounds, the substituted benzimidazoles that suppress gastric acid secretion by specific inhibition of the H+/K+ ATPase enzyme system at the secretory surface of the gastric parietal cell. The molecular formula for omeprazole is C_17_H_19_N_3_O_3_S (Figure 5). The molecular weight is 345.416.

Omeprazole was first marketed in the USA in 1989. It is now also available from generic manufacturers under various brand names. Omeprazole is available as tablets and capsules in strengths of 10, 20, 40 and 80 mg; and as a powder (omeprazole sodium) for intravenous injection. Most oral omeprazole preparations are enteric-coated, due to the rapid degradation of the drug in the acidic conditions of the stomach. In June 2004, the FDA approved an immediate-release preparation of omeprazole using sodium bicarbonate as a buffer against gastric acid degradation and no requirement for an enteric coating. This combination preparation is marketed in the USA by Santarus under the brand name Zegerid and is marketed as capsules, chewable tablets and powder for oral suspension. Zegerid is most useful for those patients who suffer from nocturnal acid breakthrough or those patients who desire immediate relief [23].

#### Pharmacodynamics

After oral administration, the onset of the anti-secretory effect of omeprazole occurs within 1 h and maximum effect occurs within 2 h. At 24 h, inhibition of secretion is approximately 50% of maximum and duration of inhibition lasts up to 72 h. Although omeprazole has a very short plasma half-life, the anti-secretory effect lasts for a long time due to prolonged binding to parietal H+/K+ ATPase enzyme. When the drug is discontinued, secretory activity returns to baseline over 3–5 days. The inhibitory effect of omeprazole on acid secretion increases with repeated once-daily dosing, reaching a plateau after 4 days. In studies involving more than 200 patients, serum gastrin levels increased during the first 1–2 weeks of once-daily administration of therapeutic doses of omeprazole in parallel with inhibition of acid secretion. Systemic effects of omeprazole in the CNS, cardiovascular and respiratory systems have not been found to date.

The delayed-release capsules are enteric-coated (as omeprazole is acid-labile) so the absorption of omeprazole begins once the granules leave the stomach. Absorption is rapid. Peak plasma levels occur within 0.5–3.5 h. The absolute bioavailability (compared with intravenous administration) of the delayed-release capsule is 30–40% at doses of 20–40 mg, due to pre-systemic metabolism. This value increases slightly when given repeatedly.

Metabolism of omeprazole is hepatic. Omeprazole is extensively metabolized by the cytochrome P450 (CYP) enzyme system. The two primary CYP isozymes involved are CYP2C19 and CYP3A4. At least six omeprazole metabolites are excreted primarily through the urine. Little, if any, unchanged drug is excreted in the urine. The half-life of delayed-release omeprazole is 0.5–1 h in healthy subjects and 3 h in those with hepatic impairment. The total clearance of the delayed-release capsule is 500–600 ml/min in healthy subjects, and the plasma clearance is 250 ml/min in geriatric populations and 70 ml/min in those with hepatic impairment. Symptoms of overdose include confusion, drowsiness, blurred vision, tachycardia, nausea, diaphoresis, flushing, headache and dry mouth.

## Phase I trials

### PA325 versus EC-ASA 325 mg

Three randomized, single-blinded studies in healthy adult volunteers tested the gastrointestinal and antiplatelet effects of ASA–omeprazole combination pills over 4 weeks. The studies varied in experimental and control dosing. The studies were designed as crossover studies to examine the pharmacokinetic properties and gastric effects of PA32520 versus EC-ASA 325 mg; PA32520 versus EC-ASA 81 mg and PA32540 versus EC-ASA 325 mg. In all studies, gastrointestinal effects were examined by endoscopy and rated according to the Lanza score, a 5-grade scoring system based upon endoscopic evaluation of the gastrointestinal mucosa (Table 1) [24].

**Table 1.** **Lanza score grading system for gastrointestinal ulceration [24]**.

**Lanza score**	**Description of gastrointestinal mucosa**
0	No visible lesions
1	Erosion or hemorrhage
2	2–10 erosions or hemorrhages
3	11–25 erosions or hemorrhages
4	>25 erosions or hemorrhages or any ulcer

The 4-week studies included only subjects with normal endoscopy at baseline (i.e., grade 0). The primary end point of the studies was the percent of patients with a Lanza score of 3 or 4 at day 28. Lanza scores were assessed at day 14 and day 28. In all studies, patients who received EC-ASA were significantly more likely to experience upper gastrointestinal damage as measured by the Lanza score and rate of ulceration. In all three studies, the combined dosing of ASA + omeprazole was associated with significant reduction in gastrointestinal damage. PA32540 displayed the greatest reduction in the rate of gastrointestinal adverse events and the lowest rate of upper gastrointestinal damage [25].

The antiplatelet effect of ASA was measured in the second study only (PA32520 vs EC-ASA 81 mg) and was measured by urinary 11-dehydrothromboxane B2 (11-dh-TXB2). High urinary 11-dh-TXB2 has been associated with an increased risk of cardiovascular events [26,27]. The change from baseline in urinary 11-dh-TXB2 was measured after 4 weeks to compare the antiplatelet and gastrointestinal effects of PA32540 with those of a lower dose EC-ASA 81 mg. PA32520 produced significantly greater inhibition of *in vivo* thromboxane generation, as measured by urinary 11-dh-TXB2, compared with EC-ASA 81 mg.

Bioequivalence measurements were taken in the third study measuring PA32540 versus EC-ASA 325 mg. This study was designed as a crossover study separated by a 5-day washout period. The rate and extent of ASA absorption of PA32540 in comparison to EC-ASA 325 mg was not altered by the combined dosing with omeprazole in PA32540 [25].

### PA32540 versus ASA 325 mg + EC omeprazole 40 mg

PA32540 was compared with EC omeprazole 40 mg in a crossover design to examine the release profile of omeprazole within PA32540 and its effect on intragastric pH. This Phase I study in 26 healthy volunteers consisted of two 7-day dosing periods separated by a washout period of 7 days or more. Pharmacokinetic testing took place on days 1, 5 and 7 of each dosing period with the primary end point of percent time intragastric pH >4 on day 7. The percent time of gastric pH >4 over 24 h on day 7 was 50.6% for PA32540 and 57.6% for EC-omeprazole. Mean intragastric pH increased more rapidly with PA32540 versus ASA + EC-omeprazole 40 mg (0.29 vs 0.60 h) due to the difference in immediate-release versus EC formulations. Total exposure to omeprazole from PA32540 was about 51–57% of that observed for EC-omeprazole 40 mg [28]. A meta-analysis of studies evaluating intra-gastric pH in healthy volunteers revealed a mean percent time intragastric pH >4 of 48.7% for omeprazole 20 mg [29]. Given that the level of acid suppression in PA32540 in this study was similar to the level of acid suppression documented in past studies of 20 mg delayed release omeprazole, it is expected that the level of gastric protection offered by omeprazole in the PA32540 formulation is sufficient [28].

## Phase III trials

Two Phase III trials were conducted in adults at risk of a gastric ulcer and with an indication for ASA therapy for secondary cardiovascular prevention. Risk was indicated by age >55 years, or a documented history of gastric or duodenal ulcer in the previous 5 years. All subjects had been taking ASA for at least 3 months but were required to have no PPI use in the 2 weeks prior to baseline endoscopy. Indications for ASA therapy varied and included past MI, stroke or TIA, a history of coronary artery bypass grafting, PCI or carotid endarterectomy or other clinically significant coronary or other atherosclerotic vascular disease. Subjects were assigned to either PA32540 or EC-ASA 325 mg and were stratified by chronic NSAID use, which they were permitted to continue during the trial. Participants were followed for 6 months, with a primary end point of endoscopically confirmed gastric ulcer, defined as a mucosal break ≥3 mm in diameter with depth.

A total of 524 subjects were followed across the two Phase III trials. At the end of 6-months, 8.6% of patients in the EC-ASA group compared with 3.2% of patients in the PA32540 group experienced a gastric ulcer (p < 0.001). These differences were apparent at 1 month (0.8 vs 3.4%; p < 0.003) and 3 months (1.7 vs 6.7%; p < 0.001).

Patients in the PA32540 group were also more likely to experience a resolution of their heartburn symptoms. At 6 months, 92.8% of patients in the PA32540 and 75.9% in the EC-ASA group experienced a resolution of their heartburn symptoms (p < 0.001). At baseline, heartburn rates were 28.4% in the PA group versus 33.5% in the EC-ASA group (p = 0.078), compared with the 6-month rates of 7.2% in the PA group and 24.1% in the EC-ASA group (p < 0.001) [30].

PA32540 patients were also less likely to discontinue their medication for any reason. The discontinuation rate due to a pre-specified upper gastrointestinal side effect was 1.5% in PA32540 users and 8.2% among EC-ASA users (p < 0.001). Among PA32540 users, 6.7% of patients compared with 11.2% of EC-ASA patients discontinued due to another adverse event (p < 0.05).

The number of Major Adjudicated Cardiovascular Events was similar across the two groups. A total of 22 events (2.1%) occurred across the treatment and control groups, including 9 (1.7%) events in the PA32540 group and 13 (2.5%) events in the EC-ASA group. The most common events were non-fatal MI (5 PA32540; 3 EC-ASA) and TIA (1 PA32540; 4 EC-ASA). One cardiovascular death occurred in the EC-ASA group [31].

The presence of gastric erosion at baseline was associated with higher rates of gastric ulceration during the trial in patients taking EC-ASA but not in those taking PA32540. Taken independently, the presence of gastric erosion at baseline and treatment assignment to EC-ASA were associated with increased chances of developing a gastric ulcer over the course of the trial. Among those who had a gastric erosion at baseline, 13% in the EC-ASA group and 4.2% in the PA32540 group developed a gastric ulcer during the trial (p = 0.001). Among those without gastric erosion at baseline, 5.9% in the EC-ASA group compared with 2.6% in the PA32540 group developed a gastric ulcer (p < 0.05). The odds ratio (OR) of developing gastric ulceration by treatment was 2.88 (95% CI: 1.62–5.12), and the OR by baseline gastric erosion was 2.12 (95% CI: 1.26–3.57) [30].

The presence of gastric erosion at baseline and assignment to EC-ASA were also associated with a higher rate of gastric erosion throughout the trial. In the EC-ASA group, 41.3% of subjects with no erosion at baseline, versus 76.5% of subjects with erosion at baseline, developed a gastric erosion (p < 0.001). In the PA32540 group, 20.7% of subjects with no erosion at baseline, versus 39.4% of subjects with erosion at baseline, developed a gastric erosion (p < 0.001).

NSAID use also increased the risk of gastric ulceration. The rates of gastric ulceration at 6 months were 4.5% for the PA32540 group and 10.2% for the EC-ASA group among those who used NSAIDs at baseline. Among non-users of NSAIDs, the rates of gastric ulceration were 3.1% in the PA group and 8.4% in the EC-ASA group [31].

## PA32540 & thienopyridines

The American Heart Association guidelines recommend a thienopyridine P2Y12 receptor antagonist in combination with ASA for treatment of patients who have undergone a stent placement after acute coronary syndrome or PCI. However, PPI dosing alongside dual antiplatelet therapy has been questioned due to concerns that it may compromise the efficacy of clopidogrel and other thienopyridines. Moreover, several studies have identified omeprazole as a particularly troublesome PPI that shows increased clopidogrel resistance in patients using this drug, based on trials comparing the effect of omeprazole with the effect of pantoprazole and famotidine on antiplatelet activity [32].

Observations of this effect have been discussed in numerous reviews, meta-analyses and retrospective studies. A retrospective cohort study of 8205 patients with ACS taking clopidogrel after hospital discharge showed a higher rate of death or rehospitalization for ACS among patients taking clopidogrel with a PPI compared with patients taking clopidogrel without a PPI (29.8 vs 20.8%; adjusted OR: 1.25; 95% CI: 1.11–1.41). However, significant confounding took place due to the fact that the PPI cohort was generally older and sicker [33]. Clinical trials, such as the CAPRIE and CREDO trials, examining the impact of PPI use in sub-analyses, have also revealed the potential for PPI to reduce the effectiveness of antiplatelet therapy [34]. Based on these concerns, the US FDA and the EMA advised in 2009 that patients taking clopidogrel should not take PPIs, even when spaced separately [35].

Despite the current guidelines, few large controlled trials have been conducted to allow for conclusive evidence to emerge. One study seeking to address this question, the COGENT trial, was stopped prematurely due to loss of funding after enrolling 3761 eligible for analysis (compared with a goal of 5000 patients). This study showed a similar event rate between the omeprazole and placebo group, with a small but significant decrease in the risk of gastrointestinal events in the omeprazole group compared with placebo [36].

A proposed mechanism of the potential interaction between clopidogrel and PPIs has been the shared reliance on CYP. Because clopidogrel is a pro-drug, it requires activation to its active metabolite through a CYP-dependent pathway. The pathway requires the CYP 2C19 isoenzyme for this conversion to occur. Some have hypothesized that the attenuated effect of clopidogrel when taken with PPIs may be due to the competitive inhibition of the CYP 2C19 isoenzyme when PPIs are administered [37]. Moreover, it has been proposed that patients who carry the loss-of-function allele of CYP2C19 polymorphism (CYP2C19*2) display significantly lower responses to clopidogrel and would be more severely impacted by any interaction with PPIs. This association, however, has been called into question in studies measuring antiplatelet response to the drug [38]. Thus, the overall understanding of the benefits and risks of combined clopidogrel and PPI use is muddled at best, with a significant lack of understanding regarding the true effects of these drugs used together and the effect of genotype on this interaction.

A non-inferiority study was conducted in healthy volunteers to measure platelet inhibition in PA32540 dosed with clopidogrel. PA32540, dosed synchronously with omeprazole in one group and spaced 10 h from clopidogrel dosing in another group, were administered to determine whether they were non-inferior to EC-ASA and clopidogrel dosed synchronously. In this study, 30 patients were randomized to receive either EC-ASA and clopidogrel followed by PA32540 and clopidogrel separated by a 14-day or greater washout period, or PA32540 and clopidogrel followed by EC-ASA and clopidogrel separated by a 14-day or greater washout period, dosed synchronously. Both groups received PA32540 and clopidogrel spaced 10 h apart after a washout period of 14 days or greater. Platelet inhibition was measured on day 1 and day 7 of each dosing period. Spaced administration of PA32540 and clopidogrel met the non-inferiority criteria, but synchronous administration did not [39].

In 2013, a similar study examined whether spacing PA32540 and clopidogrel 10 h apart would have greater antiplatelet effects than EC-ASA, clopidogrel and PPI taken together. Patients were enrolled in two 7-day treatments separated by 14-day washout periods. The treatment arms consisted of PA32540 and clopidogrel (300 mg loading/75 mg maintenance) administered 10 h later and synchronous dosing of clopidogrel, EC-ASA (81 mg) and EC omeprazole (40 mg). Inhibition of platelet aggregation was greater with spaced PA32540 + clopidogrel therapy versus synchronous clopidogrel + EC-ASA + EC omeprazole therapy (p = 0.004). There was no difference in day 7 arachidonic acid-induced aggregation. In this study, the CYP2C19 and ABCB1 genotypes were identified. Outcomes did not differ significantly by genotype [40].

One unique aspect of PA32540 is the immediate-release formulation of omeprazole in comparison with standard PPI dosing, which most commonly occurs in a delayed-release formulation. It is unclear how this might impact the interaction with clopidogrel. In summary, these spacing studies suggest that PA32540 should be used with clopidogrel only if dosing is separated.

## Regulatory

In the USA, EC-ASA and omeprazole combined tablets are not yet commercially available. A New Drug Application was filed, and the application is currently under review. In Europe, patent EP1411900 has been filed for the drug.

## Conclusion

A number of factors contribute to low ASA adherence rates measured in the population of patients prescribed the drug for secondary cardiovascular prevention. Gastrointestinal side effects and the risk of gastrointestinal bleeding may be one contributing factor. PA32540 is a coordinated delivery tablet that aims to decrease these symptoms. The ASA core, contained in a coating that dissolves in a pH >5.5 is surround by immediate-release omeprazole. Phase I trials examining the ASA + omeprazole combined tablet show similar rates of ASA absorption and similar measurements of urinary 11-dh-TXB2 in healthy volunteers, suggesting that omeprazole did not interfere with absorption or antiplatelet effects of ASA during combined dosing. Phase III results in patients at risk for a gastric ulcer based on age and past history show a similar rate of Major Adjudicated Cardiovascular Events events and a reduced incidence of gastric erosion, gastric ulceration and pre-specified gastrointestinal symptoms in the treatment group. Spacing studies of PA32540 dosed 10 h apart from clopidogrel suggest that the immediate-release omeprazole does not interfere with clopidogrel when spaced 10 h apart, which conflicts with recent studies that raised concerns about a potential clopidogrel and PPI interaction. These pre-market results suggest that PA32540 may be an option for patients on a dual antiplatelet regimen who are also at risk for severe gastrointestinal bleeding or for patients who experience negative gastrointestinal side effects from ASA therapy.

## Expert commentary

ASA is one of the most commonly prescribed cardioprotective agents in the USA today, but as discussed previously, low adherence to the drug decreases its effectiveness. ASA is not the only cardiovascular drug with suboptimal adherence, as users of statins, β-blockers and other agents also struggle to adhere to a consistent medication regimen. Introduction of a polypill has been proposed as one method of simplifying dosing to increase adherence, and early studies of the effects of this strategy, including the UMPIRE trial, appear promising. The UMPIRE trial assigned patients to either a combination of 75 mg ASA, 40 mg simvastatin, 10 mg lisinopril and 50 mg atenolol or a combination of 75 mg ASA, 40 mg simvastatin, 10 mg lisinopril and 12.5 mg hydrochlorothiazide versus usual care. In this study, adherence was 86% in the polypill groups versus 65% in the control group (relative risk of being adherent, 1.33; 95% CI: 1.26–1.41; p < 0.001). The increase in adherence was accompanied by small but significant improvements in systolic blood pressure and low-density lipoprotein-cholesterol. In the subgroup of patients with low adherence at baseline (n = 727), the discrepancy between polypill and control group adherence was even larger, with 77% of patients in the polypill group versus 23% of patients in the control group remaining adherent with the drug regimen. The results of this study suggest that among low adherers, polypharmacy may be a significant barrier to remaining on a medication regimen and that taking a single pill may reduce the burden of preventive cardiovascular medications [41].

It is unclear at this point whether combining two pills in one (thereby decreasing medications by a single pill) will increase adherence dramatically, or whether the benefits of a polypill are limited to regimens that more drastically decrease the number of pills needed. However, this preliminary evidence of the promise of the polypill, combined with additional strategies such as increased education at strategic intervention points, provide the blueprint for a strategy which has the potential to significantly improve patient adherence and prevent future morbidity and mortality resulting from cardiovascular disease. Moreover, this particular combination pill offers a targeted approach intended to improve adherence by reducing side effects in addition to simply decreasing pill count.

Since the initial Phase III trials examining concomitant versus spaced dosing of ASA and clopidogrel, additional P2Y12 inhibitors have been introduced to the market, including the oral P2Y12 inhibitors ticagrelor and prasugrel. These newer drugs appear to provide additional benefit in decreasing the risk of major cardiovascular events. A recent meta-analysis including 43,875 patients showed a decrease in major cardiovascular events from 11.56% with clopidogrel to 9.88% with the new oral P2Y12 inhibitors (OR: 0.85; 95% CI: 0.79–0.92; p < 0.0001) [42]. A subanalysis of the PLATO trial showed that patients taking higher doses of ASA (>100 mg) did not derive benefit from the use of ticagrelor compared with clopidogrel [2]. Additionally, past studies have raised concern about an increased risk of bleeding associated with these newer drugs [43,44]. The exact extent of this risk remains unclear, with the previous meta-analysis revealing no increase in major bleeding but a small increase in combined major and minor bleeding from 5.87% with clopidogrel to 6.46% with newer oral P2Y12 inhibitors (OR: 1.16; 95% CI: 1.03–1.30; p = 0.02) [42].

At this time, while PA32540 appears to be a reasonable option to help manage gastrointestinal side effects frequently caused by ASA, the extent to which the coordinated delivery tablet can address bleeding risks and gastrointestinal side effects of P2Y12 inhibitors requires further study. Although patients in the Phase III trials of PA32540 were permitted to continue their clopidogrel dosing, only a small number (n = 111) were receiving dual antiplatelet therapy in those trials and results of gastrointestinal tolerance in this subgroup were not reported [31]. Moreover, the newer P2Y12 inhibitors have not yet been tested alongside PA32540. Ticagrelor and prasugrel lack evidence of a potential interaction with PPIs and this, combined with evidence of increased potency compared with clopidogrel, makes their use appealing. These newer drugs may alleviate concerns regarding reduced effectiveness due to drug–drug interaction, particularly for patients in whom compliance with a spacing regimen is a concern. However, this potential benefit must be weighed against the possibility of an increased risk of bleeding, particularly in a population with an already elevated risk of gastrointestinal bleeding.

## Five-year view

The use of ASA as a mainstay in secondary cardiovascular prevention is unlikely to decrease in the future. The effect of ASA is so dramatic that in one meta-analysis, it was shown to contribute more to the fall in death rates in the USA than all surgical and catheter-based interventions combined [45]. Given the success of ASA in preventing cardiovascular deaths in the population at large, there is a compelling argument for sustained efforts at making the drug safer and more tolerable for a larger number of patients. The results of Phase III trials of PA32540 show the potential of a coordinated delivery pill in accomplishing these aims. This risk reduction is relevant for most populations that use ASA as a potential treatment, including those who have experienced a cardiovascular or cerebrovascular event and those who have undergone coronary artery bypass grafting.

The polypill has been hailed by some as a potential innovation in the secondary prevention of cardiovascular disease, with some calling for a reframing of the polypill as a ‘vaccine’ for cardiovascular prevention [46]. However, the potential for ASA side effects in high-risk patients has called into question the potential for ASA inclusion in such a pill. Additionally, PPI use carries its own risk of side effects, including the risk of osteoporosis, hypomagnesemia and *Clostridium difficile*-associated diarrhea. Overall, however, the results of these Phase I and III studies suggest that the combination of ASA with omeprazole significantly increases tolerance, reduces troublesome gastrointestinal side effects such as heartburn and decreases the risk of gastrointestinal bleeding and ulceration. A crucial aspect of the Phase III trials testing the safety and efficacy of PA32540 was the use of gastric ulceration as a primary end point and gastric erosion as a secondary end point, both of which were lowered considerably in the PA32540 group. In examining silent side effects in addition to the overt tolerability issues, the combination pill emerges not only as a more tolerable pill, but also as a safer alternative to ASA alone. The addition of omeprazole demonstrates that ASA may yet be a viable candidate for inclusion in a polypill for cardiovascular protection.

## Supplementary Material

Supplementary Material

## Notes

Key issues

- Adherence to aspirin or acetylsalicylic acid (ASA) therapy for secondary cardiovascular therapy remains suboptimal; gastrointestinal side effects may negatively impact adherence.
- A coordinated delivery tablet, consisting of immediate-release omeprazole surrounding an ASA core released when pH >5.5, displayed a similar rate of ASA absorption and similar inhibition of platelet aggregation as low-dose ASA alone in Phase I trials.
- Phase I and III trials of the tablets displayed similar rates of Major Adjudicated Cardiovascular Events and lower rates of gastric erosion and ulceration in healthy and high-risk subjects.
- Coordinated delivery tablets of 325 mg ASA + 40 mg omeprazole may increase tolerability, decrease risk of ulceration and bleeding and serve as one method of improving ASA adherence and reducing pill burden in patients at risk for cardiovascular disease.
