# Supplementary material for: PA32540 for the secondary prevention of cardiovascular disease in patients at risk for aspirin-associated gastric ulcers
Source: Expert Rev Cardiovasc Ther. 2014 Oct 10;12(11):1251–60. doi: 10.1586/14779072.2014.967214 (PMC4743601; doi:10.1586/14779072.2014.967214)
Supplement: Supplementary Material [file ierk_a_967214_sm0001.doc]

Appendix 1- Summary of Phase I and Phase III Outcomes

| **Phase** | **Comparison** | **Sample Size** | | **Outcomes(Primary/Secondary)** | | **Result** | |
| --- | --- | --- | --- | --- | --- | --- | --- |
| I  Gurbel et.al., 2008 | PA32520 v  EC-ASA 325 mg | 80 | | **Primary:** % of patients with a Lanza score of 3 or 4 at Day 28  **Secondary:** Incidence of gastric and/or duodenal ulcers  Effect on gastric pH of PA 325  GI tolerability | | PA32520: 7.5%  EC-ASA 325: 47.5%  P<0.001  PA32520: 0  EC-ASA 325: 20%  P=0.005  Not reported  Not reported in disaggregated form | |
| I  Alberts et.al., 2009 | PA 32520 v  EC-ASA 81 mg | 80 | | **Primary:** % of patients with a Lanza score of 3 or 4 at Day 28  **Secondary:** Incidence of gastric and/or duodenal ulcers  Gastric pH  Other GI tolerability issues  Concentration of urinary 11-d-TXB2 after 4 weeks of therapy | | PA32520: 9.8% EC-ASA 81: 20.5%  P=0.22  PA32520: 4.9%  EC-ASA 81: 5.1%  P-value not reported  Not reported  Not reported in disaggregated form  Change from baseline:  PA32520: -75.1  EC-ASA 81: -68.4  P=0.008 | |
| I  Alberts et.al., 2009 | PA 32540 v  EC-ASA 325 mg | 80 | | **Primary:** % of patients with a Lanza score of 3 or 4 at Day 28  **Secondary**: GI tolerability issues | | PA32540: 2.5%  EC-ASA 325: 27.5%  P=0.003  PA32540 (EC-ASA not reported in disaggregated form)  All GI AEs: 2 (5%)  Nausea : 1 (2.5%)  Diarrhea: 0  Abdominal pain: 1 (2.5%)  Other GI events: 3 (7.5%) | |
| I  Gurbel et.al., 2011 | PA32540 + omeprazole synchronous v PA32540 + omeprazole spaced 10 hours | | 30 | | **Primary**: To evaluate whether platelet inhibition during DAPT with PA32540 and Clopidogrel, administered synchronously or spaced 10 h apart, is noninferior to a strategy of synchronous administration of 325 mg EC-ASA and C, as defined by the upper bound of the 95% confidence interval ≤10% for the difference in LSMs of platelet inhibition between the treatments | | Upper 95% CI for difference in LSMs: Synchronous administration:13.2% IPA  Spaced administration: 9.6% IPA |
| I  Gurbel et.al., 2013 | PA32540 and clopidogrel 10 hours v  EC-ASA, clopidogrel, and PPI synchronous | | 30 | | **Primary**: Inhibition of platelet aggregation (20 μM adenosine diphosphate, maximal extent) after 7 days | | Day 7 LSM difference  7.2% (95% CI 3%-12%)  P = .004  (IPA greater with spaced administration) |
| I  Miner, et.al., 2013 | PA32540 v. ASA 325 mg + EC omeprazole 40 mg- 2013 | 36 | | **Primary**: To determine if aspirin component of PA32540 v. ASA 325 mg + omeprazole were bioequivalent ( i.e. that the 90% CI for the ratios of the geometric LSM of area under the concentration-time curve (AUC0-∞) and maximum plasma concentration (C_max_) are between 80%–125%) | | **AUC0–∞ (hr*μg/mL):**  PA32540: 104.4  EC-ASA 325: 95.4  90% CI of the geometric LSM for AUC0-∞:  1.095 (0.967, 1.239)  **C_max_ (μg/mL):**  PA32540: 17.4  EC-ASA 325: 16.1  90% CI of the geometric LSM for C_max_ :  1.077 (0.959, 1.209) | |
| III  Whellan et.al. | PA32540 v  EC-ASA 325 mg* | 530 | | **Primary**: Cumulative proportion of subjects developing endoscopically-determined gastric ulceration throughout 6 months of treatment  **Secondary**: Cumulative proportion of subjects developing endoscopically-determined gastric and/or duodenal ulcers at 6 months  Proportion of subjects with “Treatment Success”**  Discontinuations due to pre-specified upper GI adverse events  Heartburn resolution | | PA32540: 3.2%  EC-ASA 325: 8.6%  P<0.001  PA32540: 3.4%  EC-ASA 325: 11.6%  P<0.001  PA32540: 95.2%  EC-ASA 325: 83.2%  P<0.001  PA32540: 1.5%  EC-ASA 325: 8.2%  P<0.001  Baseline heartburn rate:  PA32540: 28.4%  EC-ASA 325: 33.5%  P=0.078  Month 6 heartburn rate:  PA32540: 7.2%  EC-ASA 325: 24.1%  P<0.001 | |
| III  Whellan et.al. | PA32540 v  EC-ASA 325 mg* | 519 | | Same as above | |

LSM= Least squared means; EC-ASA= Enteric coated aspirin

*Study data is available only in aggregated form.
** Defined as those subjects without gastric ulcers and without pre-specified upper GI adverse events leading to study discontinuation
